# Repair of aortoesophageal fistula due to a penetrating atherosclerotic ulcer of the descending thoracic aorta and literature review

**DOI:** 10.1186/1749-8090-2-12

**Published:** 2007-02-14

**Authors:** Guruvegowda Chandrashekar, Vijay MN Kumar, Ashok K Kumar

**Affiliations:** 1 Department of Cardiothoracic Surgery, Mallya Hospital, Bangalore 560001, India; 2 Department of Cardiothoracic Surgery, Patiala Heart Institute, Patiala 147001, India; 3 Department of Gastroenterology, Mallya Hospital, Bangalore 560001, India

## Abstract

Penetrating atherosclerotic ulcer rupturing into the esophagus is rare and the resulting aortoesophageal fistula carries a high mortality. In view of the emergency nature of the entity and complexity of the procedure management of such a condition is not standardized. The immediate concern is to save the patient from life threatening exsanguinations. Contrary to the practice hitherto followed no active surgical intervention was carried out for the esophageal lesion and cardiopulmonary bypass support was not employed. We present a case of rupture of a penetrating atherosclerotic ulcer of descending thoracic aorta, where in an emergency surgery was performed and the patient is doing well 21 months later.

## Case report

A 60-year old male was admitted in the emergency ward with the history of massive hematemesis the previous night. Patient had difficulty in swallowing and retrosternal discomfort for the past 2 months for which he was being treated. Once in the hospital there was no fresh bout of hematemesis. On admission the patient's pulse rate was 124/minute and blood pressure was 100/70 mmHg. Hemoglobin level was 9.9 g/dl and the white blood cell count was 13,200/cmm. The chest X-ray showed bilateral emphysematous bullae. Upper GI endoscopy showed esophageal ulceration at 32 cm.

**Figure 1:**
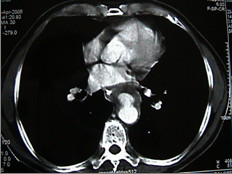
Contrast enhanced CT scan showing aortoesophageal fistula.

**Figure 2:**
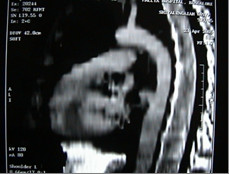
The size of the aorta appears normal on saggital reconsruction.

**Figure 3:**
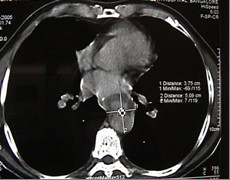
Contrast enhanced CT scan showing psedo-aneurysm formation.

Contrast enhanced chest CT images acquired at thoracic region showed a penetrating aortic ulcer into the thoracic esophagus and focal anterior aortic defect with pseudo aneurysm formation [Fig 1, 2, 3]. There was no pleural effusion. A diagnosis of aortoesophageal fistula due to penetrating aortic ulcer was made and the patient taken up for an emergency surgery.

**Figure 4:**
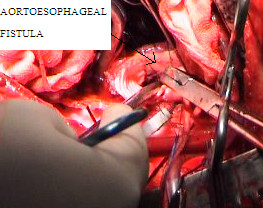
Intraoperative colour photograph showing the lesion at the end of the needle holder.

**Figure 5:**
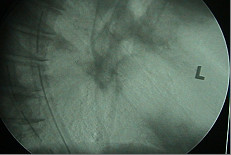
Post-operative esophagography.

**Figure 6:**
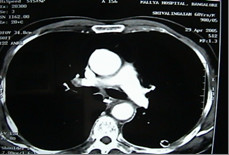
Post-operative contrast CT showing complete healing of the lesion.

He was intubated with double lumen endotracheal tube. Left radial and dorsalis pedis arteries were cannulated for the arterial pressures monitoring of upper and lower body respectively. A pulmonary artery catheter was introduced. An epidural catheter was placed to facilitate cold saline irrigation of the epidural space during aortic cross clamping. Injection Methylprednisolone 1 G was given intravenously for spinal cord protection. While securing of the femoral artery and vein in the left groin was being done, thoracotomy was performed through a posterolateral incision. There was no hematoma or effusion or pus in the pleural cavity. Descending thoracic aorta was enlarged but not to aneurismal proportions [Fig 3], the medial portion of the lower lobe of the left lung was adherent to the esophagus in the region of the fistula. Aorta was mobilized from the left subclavian artery to the diaphragm except for the area with dense adhesions that contained the fistula. Heparin was given at 1 mg/Kg and after obtaining an ACT of > 400 seconds and aortic cannulation was done distal to the left subclavian artery and also the left femoral artery cannulated using appropriate arterial cannulae and were connected by an 1/4^th ^inch polyvinyl chloride [PVC] tube establishing an aortofemoral shunt. The aorta was cross-clamped and the intercostals arteries were controlled with micro bulldog clamps. The distal aortic pressure maintained between 60 to 70 mmHg. Aorta was opened with a longitudinal incision and the aneurismal mouth was seen in the anteromedial aspect measuring 3 cm × 2 cm in size and oval in shape [Fig 4]. The base of the ulcer was filled with clots. Rest of the aorta appeared normal and edge of the defect appeared healthy. The defect was closed with a gelatin sealed Dacron vascular prosthesis [Vascutek Gelweave woven Dacron graft manufactured by Sulzer Vascutek USA, Inc., Austin, TX78752] cut into an oval shape measuring 3 cm × 3 cm using 4/0 polypropylene continuous suture technique. The aortotomy was closed with 4/0 polypropylene sutures reinforced with Teflon felt on either side. Aortic cross-clamp was released and aortofemoral shunt clamped. The total cross-clamp time was 65 minutes. Heparin was reversed with protamine. Decannulation was performed and the femoral artery repaired with 6/0 polypropylene continuous sutures. Due to dense adhesions present between the pseudoaneurysm, the esophagus and the hilum of the left lung, further dissection of the esophagus was abandoned. A Ryle's tube was introduced and its' position in the distal esophagus confirmed by palpation. Chest and the mediastinum were copiously irrigated and drained with two 36 F chest tubes. Patient maintained stable haemodynamics with adequate urine output and was able to move both lower limbs. He was kept nil orally for the next 5 days with continuous Ryle's tube aspiration. Total parenteral nutrition was begun in the immediate postoperative period and he was maintained on peri-operative antibiotic coverage of Vancomycin, cefpirome, Metranidazole and Amikacin. The per-operative culture from the thoracic cavity revealed no growth of organisms 48 hours later. On the sixth postoperative day gastrograffin esophagography revealed no esophageal leak. A contrast enhanced CT scan of the chest revealed neither aortic nor esophageal leak [Fig. 5, 6]. Patient was encouraged to take sips of sterile water.

The patient was afebrile, tolerating soft diet and ambulatory on the ninth postoperative day when he was discharged from the hospital on an antihypertensive drug.

## Discussion

Eighty-seven percent of aortoesophageal fistulas (AEFs) [1] were the result of a thoracic aortic aneurysm, foreign body ingestion, or esophageal malignancy. The remaining cases of AEF were caused by surgical complication, esophageal reflux, traumatic false aneurysm, tuberculosis, corrosive esophagitis, congenital anomaly, instrumentation, or atherosclerotic disease. Only two cases of a penetrating atherosclerotic aortic ulcer rupturing into the thoracic esophagus have been reported [2] and ours is the second such report. Since the time Snyder et al [3] reported first survival of a case of aortoesophageal fistula due to a thoracic aortic aneurysm in 1983 few long time survivors have been reported. The triad of mid thoracic pain, sentinel arterial hemorrhage, and final exsanguination after a symptom free interval has been termed as Chiari's triad [4]. The latent interval between prodromal hemorrhage and exsanguination is unpredictable and ranges from 2 hours to 18 days (mean, 2 to 3 days) [5]. We operated on the patient within hours after the sentinel hemorrhage. A differential diagnosis considering the possibility of a thoracic aortic aneurysm should always be required in patients with dysphagia. Emergency surgery should always be performed in patients showing three characteristic symptoms (hematemesis, dysphagia, chest pain) before the occurrence of massive hemorrhage [6]. A definitive diagnosis can be established when ulceration accompanied by coagulations are detected by endoscopy. Contrast enhanced CT scan confirms the diagnosis [Fig 1, 2, 3] [6] CT scan showed pseudoaneurysm but no pneumatization. On account of high probability of death from exsanguination or from infection of the surrounding tissues and subsequent sepsis, an aggressive treatment without delay has been advised [6].

There is little consensus about the optimal management of the condition. Several types of treatment have been described which include open surgery, temporary control measures such as percutaneous embolization, and the use of a Sengstaken-Blakemore tube and more recently endovascular treatment [7]. The size of the aorta being normal in our case [Fig. 3] an oval patch of Vascutek Gelweave woven Dacron graft was employed to close the defect from inside similar to the technique adopted by Fuyuhiko yasuda etal [4]. DeSilva etal [7] reviewed eleven successfully repaired cases of aortoesophageal fistulas. The treatment modality adopted varies between simple primary closures at one end of the spectrum to aggressive esophagectomy followed by reconstruction. The esophagus was repaired by primary suture in seven, except in one patient in whom the esophagic lesion was not found. Among them six patients developed recurrences of AEF or disruption of the esophageal closure and one developed paraplegia. Hence he concluded that the primary suturing of the esophagus has high recurrence rate due to disruption and the mortality is high. The esophageal blood supply is predominantly segmental in nature and any excessive dissection around the esophagus may get further devitalized and the surgeon gets committed to esophagectomy lest the danger of disruption and sepsis. We did not dissect the esophagus due to dense adhesions and this may have helped to preserve arterial blood supply enabling the esophagus to heal naturally helped by the small size of the rent. The esophageal perforation was treated conservatively and its complete healing was demonstrated by gastrograffin swallow and repeat contrast-enhanced CT scan of the chest [Fig. 5, 6]. As per Michael Reardon etal [8], in these critically ill patients the goal is to keep the surgical procedure as simple and swift as possible and avoid loss of the esophagus through resection, as all forms of esophageal substitution are inferior to native esophagus. Appropriate antibiotic coverage, copious irrigation of the pleural cavity may prevent recurrence of infection and graft rejection.

Patients with AEFs and clinical signs of infection who are in critical physical condition making them high risk candidates for open surgery should be considered for endovascular surgery as a palliative therapy or a temporary alternative till they are well enough to tolerate open surgery. It is noteworthy that six patients under the endovascular procedure group were doing well at follow-up that ranged from 6 months to 36 months [7].

The closure of the aortic defect with a Dacron patch or a tube graft without disturbing the esophageal component of the fistula is akin to endovascular graft placement. In the endovascular procedure group the esophageal component was treated conservatively or ressected at a later date when the general condition of the patient improved.

Since most surgical corrections were performed in emergencies [7] adjuncts like aortofemoral shunt [or Gott shunt] [9] and epidural space irrigation for avoiding neurological sequelae could not be applied. The general physical condition of the patient was stable in the absence of any fresh bout of hematemesis hence methods already mentioned were used to protect the spinal cord and circulation was maintained below diaphragm [10].

In conclusion the primary procedure must be directed at preventing death from exsanguinations and should be undertaken within the latent period. If there are no overt signs of sepsis and in the presence of dense adhesions the esophageal resection could be delayed or even avoided as in this case. A passive aortofemoral shunt instead of a partial or total CP bypass is an alternative in a stable patient and reduces the morbidity associated with extra corporeal circulation. However, in the absence of a large series of such cases it is not possible to be rigid about any one particular modality of treatment. The management of the esophageal pathology should be individualized based on the extent of esophageal destruction.

## Abbreviations

AEF = Aortoesophageal fistula

ACT = Activated Clotting Time

PVC = Poly Vinyl Chloride

CP = Cardio Pulmonary
